# Serum Inflammatory Markers and Amputations in Hand Osteomyelitis: A Retrospective Review of 146 Cases

**DOI:** 10.1177/15589447211066346

**Published:** 2022-02-08

**Authors:** Matthew Wyman, Dallan Dargan, Diana Kazzazi, Jennifer Caddick, Victoria Giblin

**Affiliations:** 1 Sheffield Teaching Hospitals NHS Foundation Trust, UK; 2 The University of Sheffield, UK

**Keywords:** osteomyelitis, hand, C-reactive protein, bone infection, amputation

## Abstract

**Background:**

The diagnosis of hand osteomyelitis requires correlation of clinical, radiological, and microbiological findings. The role of serum inflammatory markers in diagnosing and prognosticating hand osteomyelitis remains uncertain. We sought to determine the utility of inflammatory markers in the diagnosis and follow-up of hand osteomyelitis, and their ability to predict outcomes, particularly amputation.

**Methods:**

We retrospectively reviewed 146 patients diagnosed with hand osteomyelitis and with serum inflammatory marker levels measured after the onset of symptoms and within 14 days either side of diagnosis. Blood results at first presentation including white cell count (WCC), neutrophil-lymphocyte ratio (NLR), platelet-lymphocyte ratio (PLR), and C-reactive protein (CRP) were reviewed, and associations with amputations assessed. Follow-up markers taken at 15 to 60 days from diagnosis were analyzed where available.

**Results:**

Mean WCC and CRP at diagnosis were 9.2 (SD: 4.6) and 30.2 (SD: 42.4) respectively, compared with 8.2 (SD: 3.9) and 30.2 (SD: 42.4) at follow-up. At diagnosis, sensitivity of CRP was 74%, and WCC was 31%. Each marker had a low positive predictive value for amputation at diagnosis (<29%). A rise in CRP between diagnosis and follow-up was associated with an increased risk of amputation compared with a fall in CRP. The finding that WCC and CRP were both normal at diagnosis had a high negative predictive value against amputation (96%).

**Conclusion:**

C-reactive protein has a higher sensitivity than WCC, NLR, and PLR when used as a diagnostic adjunct in hand osteomyelitis. White cell count and CRP both within reference ranges at diagnosis was highly negatively predictive against amputation.

## Introduction

Osteomyelitis of the hand occurs relatively uncommonly compared with osteomyelitis at other sites of the body.^1^ Nevertheless, it can result in substantial disability from bony destruction and subsequent surgical debridement, and therefore, timely diagnosis is important to reduce morbidity.^2,3^ In osteomyelitis of the foot, serum C-reactive protein (CRP) has been shown to have a sensitivity and specificity of >70% when used as an adjunct to diagnosis,^4-6^ and is also valuable in evaluating treatment efficacy,^7,8^ while white cell count (WCC) is less effective as a prognostic marker in foot osteomyelitis than CRP.^9,10^

Values for the neutrophil-lymphocyte ratio (NLR) and platelet-lymphocyte ratio (PLR) can be readily calculated from a standard full blood count or complete blood count analysis (neutrophil count/lymphocyte count, and platelet count/lymphocyte count). Neutrophil recruitment, prothrombotic activity, and lymphocytic apoptosis are common observations in systemic inflammation, resulting in neutrophilia, thrombocytosis, and lymphopenia, and thus increasing the NLR and PLR.^11,12^ Both NLR and PLR predict prognosis in critically ill patients, and in a range of conditions including Alzheimer disease, coronary heart disease, and COVID-19.^13-15^

The diagnosis and monitoring of response to treatment of hand osteomyelitis are unique compared with osteomyelitis at other sites, yet existing evidence-based guidelines are tailored to long bones, for instance, recent fracture-related infection guidelines,^16^ and guidelines for the investigation of bone and soft tissue associated with osteomyelitis,^17^ so it is unclear how much they can be extrapolated to hand osteomyelitis.

Reilly et al^18^ studied 46 patients with hand osteomyelitis and noted no correlation between erythrocyte sedimentation rate (ESR) and final outcome, and no marked trends in WCC among patients with hand osteomyelitis. A series of bacterial hand infections has shown a normal CRP in 131/208 patients (63%); however, CRP did not distinguish between soft tissue infection and osteomyelitis.^19^ Other studies which have focused primarily on septic arthritis of the hand rather than short bone osteomyelitis have shown that septic arthritis in small joints of the hand has lower CRP values compared with larger joints, mean: 59 mg/L (SD: 78) versus mean: 156 mg/L (SD: 107).^20^

We sought to determine the utility of inflammatory markers in the diagnosis and follow-up of hand osteomyelitis. We additionally studied the ability of inflammatory markers to predict outcomes, particularly amputation.

## Materials and Methods

### Patients

A retrospective review of patients with hand osteomyelitis was performed as part of a project approved by our institutional review board (STH 20214). Radiology reports, operative records, and daily trauma handover lists over a 12-year period (2008-2019) in a tertiary referral center were screened for potential cases of osteomyelitis of the phalanges and metacarpals of the hand. The clinical records of all possible cases were reviewed to confirm a diagnosis of osteomyelitis. Those with a positive clinical diagnosis and serum inflammatory marker measurements performed at the time of diagnosis were considered eligible for inclusion.

### Diagnosis of Osteomyelitis

Diagnosis of metacarpal or phalangeal osteomyelitis was based upon a combination of microbiological cultures, clinical presentation, and radiograph imaging. The date of diagnosis of osteomyelitis was retrospectively established for each patient based on the date of the first positive bone biopsy. If no bone biopsy was available, the first radiograph with bone changes consistent with osteomyelitis was taken as the date of diagnosis. For patients with recurrent osteomyelitis, only the first episode of osteomyelitis was considered. Cases treated as conditions other than osteomyelitis by a hand surgeon throughout their treatment course were excluded.

### Laboratory Analysis

Laboratory records were screened for inflammatory marker measurements. Patients were considered eligible if “diagnostic inflammatory markers” were available. These were defined as serum inflammatory marker measurements (at least one of WCC, NLR, PLR, or CRP) taken after the onset of symptoms and within 14 days either side of diagnosis. If serial samples were performed within this “diagnostic window,” the inflammatory markers measured closest to the date of diagnosis were included in the analysis. Patients with osteomyelitis but without any available serum inflammatory marker measurements, or with measurements falling outside the diagnostic window, were excluded.

“Follow-up inflammatory markers” were defined as inflammatory markers measured 15 days or more after the date of diagnosis. If serial follow-up samples were obtained, the inflammatory markers measured closest to 21 days from the date of diagnosis were recorded for analysis, as this marked the mid-point of a 6-week course of antibiotics. Inflammatory markers measured more than 60 days after diagnosis were excluded as it was anticipated that by this time, resolution of infection would have been achieved in a majority of cases.

The NLR was calculated by dividing the neutrophil count by the lymphocyte count; PLR was calculated by dividing the platelet count by the lymphocyte count. Reference ranges for WCC (3.5-9.5 × 10^9^) and CRP (0-5 mg/L) were those used by the laboratory at our center. Reference ranges for NLR (0.78-3.53) and PLR (61-221) were based on the findings of population studies investigating the normal ranges of these markers among healthy Caucasian adults.^21,22^ Markers were considered elevated if they were equal to or greater than the upper limit of the normal reference range, in keeping with our laboratory protocols.

### Statistical Analysis

Normally distributed variables were described as mean and standard deviation (SD), and range. Nonparametric data were described as median and range. Sensitivity, specificity, and positive and negative predictive values were assessed for diagnosis and amputation outcomes. Statistical comparison between included and excluded patients was performed using Pearson χ^2^ test.

## Results

In the 12-year period, from 2008 to 2019, 210 individuals were diagnosed with osteomyelitis of the phalanges or metacarpals of the hand. Of 210 patients, 146 (70%) met the inclusion criteria, and were eligible for inclusion. The demographic and clinical characteristics of the study population are represented in Table 1.

**Table 1.** Demographic Characteristics of 146 Patients With Hand Osteomyelitis and Inflammatory Markers at Diagnosis.

Characteristics of population	Values for each parameter
Age at diagnosis (mean, SD), y	59.9 (16.8)
Male/female (%)	101 (69) / 45 (31)
Type 1/type 2 diabetes (%)	12 (9) / 40 (27)
CKD stage 5 (%)	22 (15)
eGFR (mean, SD)	65.8 (29.2)
Digital artery calcification (%)	28 (19)
History of tobacco smoking (%)	61 (42)
History of intravenous drug use (%)	7 (5)
Median bones with osteomyelitis (range)	1 (1-25)
Median operative procedures required (range)	1 (0-30)
Phalanx amputation^a^ (%)	44 (30)
Digit amputation^b^ (%)	19 (13)
All-cause mortality at 1 y; n (%)	18 (12)

*Note.* CKD = chronic kidney disease; eGFR = estimated glomerular filtration rate.

a Amputation of one or more phalanx without complete amputation of the digit.

b Amputation of entire digit, with or without ray amputation.

Diagnostic inflammatory markers were measured at a median of 1 day from clinical diagnosis (range: 0-14 days). Inflammatory markers were measured up to 14 days before the date of diagnosis in 37 cases (25%), on the date of diagnosis in 58 cases (40%), and within 14 days after the date of diagnosis in 51 cases (35%). Follow-up markers were collected for 68 (47%) patients at 15 to 60 days from diagnosis. The number of patients with raised inflammatory markers at diagnosis and follow-up is represented in Table 2.

**Table 2.** Proportion of Patients With Elevated Inflammatory Marker Measurements, at Diagnosis and at Follow-up.

Inflammatory marker (cut-off)	Diagnosis markers (−14 to 14 days from diagnosis)			Follow-up markers (15 to 60 days from diagnosis)			Diagnosis vs follow up
	No. tests at diagnosis No. with marker elevated (%)/not elevated (%)			No. tests at follow-up No. with marker elevated (%)/not elevated (%)			OR, 95% CI
Total no.	n = 146			n = 68			—
WCC (≥9.5 × 10^9^)	n = 144 45 (31) / 99 (69)			n = 67 20 (30) / 47 (70)			OR: 1.07, 95% CI, 0.57-2.01
NLR (≥3.53)	n = 144 62 (43) / 82 (57)			n = 67 29 (43) / 38 (57)			OR: 0.99, 95% CI, 0.55-1.78
PLR (≥221)	n = 144 41 (28) / 103 (72)			n = 67 21 (31) / 46 (69)			OR: 0.87, 95% CI, 0.46-1.64
CRP (≥5 mg/L)	n = 121 89 (74) / 32 (26)			n = 51 33 (65) / 18 (35)			OR: 1.51, 95% CI, 0.75-3.06
Combined markers	Both elevated (%)	Either elevated (%)	Neither elevated (%)	Both elevated (%)	Either elevated (%)	Neither elevated (%)	OR, 95% CI (both elevated at diagnosis vs both elevated at follow-up)
WCC and CRP		n = 119		n = 50			OR: 1.16, 95% CI, 0.56-2.41
	37 (31)	56 (47)	26 (22)	14 (28)	22 (44)	14 (28)	

*Note.* No. = number; n = number of patients; ref range = reference range; WCC = white cell count; NLR = neutrophil-lymphocyte ratio; PLR = platelet-lymphocyte ratio; CRP = C-reactive protein; OR = odds ratio; CI = confidence interval.

Mean WCC and CRP at diagnosis were 9.2 (SD: 4.6) and 40.5 (SD: 53.9) respectively, compared with 8.2 (SD: 3.9) and 30.2 (SD: 42.4) at follow-up. Mean NLR and PLR at diagnosis were 4.4 (SD: 3.5) and 194.9 (SD: 121.7) respectively, compared with 4.1 (SD: 3.2) and 198.0 (SD: 150.3) at follow-up. One patient had a WCC >20 × 10^9^ at diagnosis as a result of sepsis, and 2 patients had WCC >20 × 10^9^ at both diagnosis and follow-up as a result of preexisting acute myeloid lymphoma and myelofibrosis. Fourteen patients with CRP at diagnosis (12%) had marked elevation (>100 mg/L); 11 of these patients had hand cellulitis, including 7 with systemic inflammatory responses consistent with sepsis.

Sixty-four patients (44%) required digit shortening. The entire digit or ray was amputated in 19 (30%), hereafter grouped as “digit amputation,” while the remaining 45 (70%) required amputation of 1 or more phalanges (hereafter grouped as “phalanx amputation”). Table 3 represents the rates of phalanx and digit amputation among patients with a rise in inflammatory markers between diagnosis and follow-up.

**Table 3.** Phalanx and Digit Amputation Rates for Patients With a Rise in an Inflammatory Marker Between Diagnostic and Follow-up Measurements.

Inflammatory marker	Change in inflammatory marker	Phalanx amputation^a^ (%)	Digit/ray amputation^b^ (%)	Any amputation (%)	OR, 95% CI Higher vs equal or lower at follow-up, comparison of amputation	*P* value Higher vs equal or lower at follow-up, comparison of amputation
WCC	Higher at follow-up, n = 25	9 (36)	6 (24)	15 (60)	OR: 2.0, 95% CI, 0.73-5.47	.17 χ^2^
	Equal or lower at follow-up, n = 42	9 (21)	9 (21)	18 (43)		
NLR	Higher at follow-up, n = 26	9 (35)	5 (19)	14 (54)	OR: 1.4, 95% CI, 0.50-3.62	.55 χ^2^
	Equal or lower at follow-up, n = 41	9 (21)	10 (24)	19 (46)		
PLR	Higher at follow-up, n = 23	7 (30)	5 (22)	12 (52)	OR: 1.2, 95% CI, 0.44-3.3	.73 χ^2^
	Equal or lower at follow-up, n = 44	11 (25)	10 (23)	21 (48)		
CRP	Higher at follow-up, n = 11	6 (55)	4 (36)	10 (91)	OR: 12.7, 95% CI, 1.45-110.3	<.01 FE
	Equal or lower at follow-up, n = 34	7 (21)	8 (24)	15 (44)		

*Note.* WCC = white cell count; NLR = neutrophil-lymphocyte ratio; PLR = platelet-lymphocyte ratio; CRP = C-reactive protein; OR = odds ratio; CI = confidence interval; FE = Fisher exact test.

a Amputation of one or more phalanx without complete amputation of the digit.

b Amputation of entire digit, with or without ray amputation.

The sensitivity of inflammatory markers as a diagnostic adjunct in osteomyelitis of the hand, and the sensitivity, specificity, and predictive value of diagnostic inflammatory markers in predicting either phalanx amputation or digit amputation, are represented in Table 4.

**Table 4.** Sensitivity, Specificity, and Predictive Values of Inflammatory Markers in Diagnosis and Predicting Amputation in Cases of Osteomyelitis of the Hand.

Inflammatory marker	Sensitivity (%)	Specificity (%)	Positive predictive value (%)	Negative predictive value (%)
Elevated WCC (≥9.5 × 10^9^)				
Diagnosis	31	—	—	—
Phalanx amputation^a^	39	75	55	61
Digit/ray amputation^b^	47	71	20	90
Elevated NLR (≥3.53)				
Diagnosis	43	—	—	—
Phalanx amputation	54	65	55	65
Digit/ray amputation	68	60	21	93
Elevated PLR (≥221)				
Diagnosis	28	—	—	—
Phalanx amputation	37	78	56	61
Digit/ray amputation	63	77	29	93
Elevated CRP (≥5 mg/L)				
Diagnosis	74	—	—	—
Phalanx amputation	85	37	53	75
Digit/ray amputation	94	33	26	96
Elevated WCC or CRP				
Diagnosis	82	—	—	—
Phalanx amputation	85	29	52	69
Digit/ray amputation	94	25	18	96

*Note.* WCC = white cell count; NLR = neutrophil-lymphocyte ratio; PLR = platelet-lymphocyte ratio; CRP = C-reactive protein.

a Amputation of one or more phalanx.

b Amputation of entire digit, with or without ray amputation.

Compared with the study population (n = 146), the excluded individuals (n = 64) were broadly similar and had nonsignificant differences in rates of phalanx amputation (30% of included patients vs 23% of excluded patients [relative risk: 0.91, *P* = .32]), digit amputation (13% of included patients vs 6% of excluded patients [relative risk: 0.82, *P* = .15]), and all-cause mortality at 1 year (12% of included patients vs 6% of excluded patients [relative risk: 0.83, *P* = .18]).

## Discussion

This study shows that CRP is more sensitive than the other inflammatory markers evaluated when used as a diagnostic adjunct (74%) and only a minority of patients with hand osteomyelitis (45/144, 31%) have raised WCC at the time of diagnosis, consistent with another series of hand osteomyelitis.^18^

Likewise, NLR and PLR had low sensitivities as diagnostic adjuncts for hand osteomyelitis (43% and 28%, respectively). The relative insensitivity of these markers may be a reflection that osteomyelitis within the small bones of the hand may generate a localized rather than systemic inflammatory response, which would lead to increased WCC, NLR, and PLR. Our study found 14 patients had CRP >100 mg/L at diagnosis, which is a sensitive and specific cut-off for sepsis in critical care populations,^23,24^ and 7 of these 14 patients had sepsis.

In a subgroup of 7 patients in our study with hand osteomyelitis, rheumatological conditions, and secondary Raynaud phenomenon, CRP was not universally elevated.^25,26^ Numerous studies have found CRP to be more sensitive than WCC when used to identify bacterial infection,^27-29^ and that even minor infections such as small colonized wounds can cause an increase in CRP,^30^ which may explain increased sensitivity relative to other inflammatory markers.

Inflammatory markers appeared to trend downward in most patients with follow-up measurements available, which is a trend also noted in serial inflammatory marker measurements in foot osteomyelitis.^8^ Between diagnosis and follow-up, we identified a small decrease in mean WCC (9.2 vs 8.2) and mean CRP (40.5 vs 30.2). Patients with a rise in their serum inflammatory marker measurements between diagnosis and follow-up underwent phalanx and digit amputation at higher rates than the overall study population; in particular, those whose CRP rose between diagnosis and follow-up were significantly more likely to require amputation than those whose CRP remained static or fell (10/11 vs 15/34, *P* < .01). However, it was noted that follow-up WCC, NLR, and PLR were elevated in 30%, 43%, and 31% of patients, respectively, which are similar to the proportion of patients with elevated markers at diagnosis. This may limit the utility of these markers to monitor disease progression.

Each of the serum inflammatory markers was more likely to be elevated at the time of diagnosis among patients who later required amputation, suggesting that severe bone infections requiring amputation generate a larger inflammatory response. The sensitivity of CRP >5 mg/L was 85% among patients requiring amputation of at least 1 phalanx, and 94% among patients requiring complete digit amputation. Among patients requiring complete amputation, both NLR and PLR had reasonable sensitivity (68% and 63%, respectively) and specificity (60% and 77%, respectively). The negative predictive value for complete digit amputation was ≥90% for each inflammatory marker; however, the positive predictive value was poor (<29% for each marker).

Confounding variables, including diabetes mellitus and peripheral vascular disease, which are associated with systemic inflammation and elevated inflammatory markers even in the absence of infection, were prevalent among patients requiring amputation (33/64 [52%] and 15/64 [23%] respectively).^31-34^ Our study found that 43 of the 48 patients (90%) with either diabetes mellitus or end-stage renal failure had elevated CRP at diagnosis, compared with 46/73 (63%) without.

Erythrocyte sedimentation rate has been shown to be raised in foot osteomyelitis compared with soft tissue infection alone (76.2 mm/h, SD: 34.6 vs 59.2, SD: 24.7),^35^ however was performed in only 26/146 patients (18%) in this series and hence no analysis was conducted. Serum procalcitonin, although not used in this study, is a sensitive and specific inflammatory marker in distinguishing acute gout from bacterial infection (sensitivity and specificity of 81%)^36^ and should be considered when gout remains a differential diagnosis, although it is a less useful diagnostic marker for bacterial infection in patients with chronic gout (sensitivity: 22%, specificity: 62%).^37^

The retrospective nature of this study, along with a lack of a control group with other hand infections or inflammatory conditions, are limiting factors. We cannot comment on the specificity, positive predictive value, or negative predictive value of inflammatory markers at diagnosis of hand osteomyelitis. Sixty-four were excluded because of a lack of serum inflammatory marker measurements.

Prospective studies investigating inflammatory markers in the context of osteomyelitis often present measurements taken within 48 hours of diagnosis,^8,33,35^ although the date of diagnosis does not always correspond to a fixed point in the clinical course, and plain radiograph evidence may be absent for 10 to 14 days from disease onset. Efforts were made to standardize the time frame within which inflammatory markers were measured; however, there was heterogeneity in timing of collection of markers at diagnosis and follow-up. Most patients with diagnostic inflammatory marker measurements (53%) had no subsequent measurements available, preventing us from making an assessment of the utility of these markers in the monitoring of hand osteomyelitis. However, studies looking at osteomyelitis in other bodily sites have shown that CRP trends downward with successful treatment, and so despite the limited follow-up data available in our study, we believe that a rise in CRP between diagnosis and follow-up indicates unsuccessful treatment and is therefore a significant predictor of amputation.^7,8^

The lack of published series and evidence-based guidelines means that inflammatory markers do not yet have a well-defined role in cases of hand osteomyelitis. This has led to inconsistency in serum inflammatory marker measurements for patients in this series, as well as other aspects of individual patient management which may have varied across the 12 years analyzed. Owing to varied sensitivity of inflammatory markers and inconsistency of results between patients, their role at present remains secondary to bone biopsy, imaging and clinical, and intraoperative findings in most cases. At a local level, our center has introduced a set of guidelines which includes the routine monitoring of serum inflammatory markers at the time of diagnosis and at subsequent follow-up appointments, which will enable further research in this field. Prospective studies with regimented inflammatory marker measurements, including ESR and procalcitonin, in similar cohorts, are needed to better assess the role of serum inflammatory markers in hand osteomyelitis.

In conclusion, C-reactive protein was the most sensitive marker for hand osteomyelitis, but was not universally elevated, and CRP >100 mg/L was associated with sepsis. White cell count, NLR, and PLR were of limited utility in this series. An increase in CRP between diagnosis and follow-up was associated with an increased risk of amputation. If neither CRP nor WCC were elevated at diagnosis, the risk of subsequent amputation was low (4%).
